# DIPS-Plus: The enhanced database of interacting protein structures for interface prediction

**DOI:** 10.1038/s41597-023-02409-3

**Published:** 2023-08-03

**Authors:** Alex Morehead, Chen Chen, Ada Sedova, Jianlin Cheng

**Affiliations:** 1 University of Missouri, Electrical Engineering & Computer Science, Columbia, MO 65211 USA; 2 Oak Ridge National Laboratory, Oak Ridge, TN 37830 USA

**Keywords:** Machine learning, Scientific data

## Abstract

In this work, we expand on a dataset recently introduced for protein interface prediction (PIP), the Database of Interacting Protein Structures (DIPS), to present DIPS-Plus, an enhanced, feature-rich dataset of 42,112 complexes for machine learning of protein interfaces. While the original DIPS dataset contains only the Cartesian coordinates for atoms contained in the protein complex along with their types, DIPS-Plus contains multiple residue-level features including surface proximities, half-sphere amino acid compositions, and new profile hidden Markov model (HMM)-based sequence features for each amino acid, providing researchers a curated feature bank for training protein interface prediction methods. We demonstrate through rigorous benchmarks that training an existing state-of-the-art (SOTA) model for PIP on DIPS-Plus yields new SOTA results, surpassing the performance of some of the latest models trained on residue-level and atom-level encodings of protein complexes to date.

## Background & Summary

**Fig. 1:**
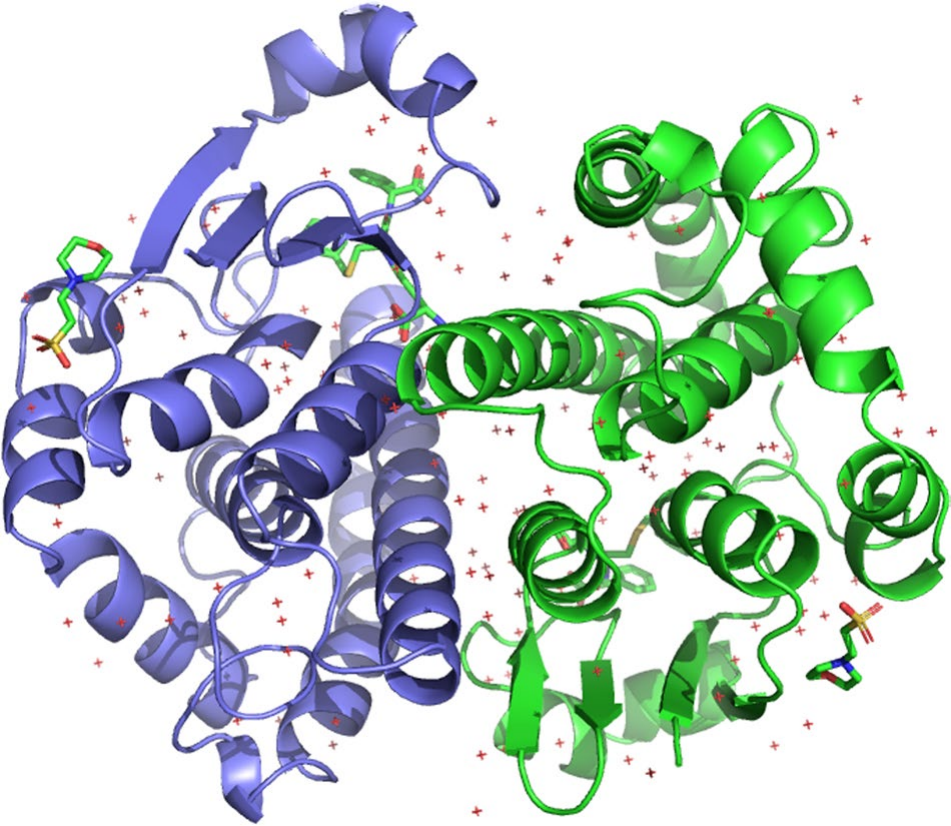
A PyMOL^48^ visualization for a complex of interacting proteins (PDB ID: 10GS).

Proteins are one of the fundamental drivers of work in living organisms. Their structures often reflect and directly influence their functions in molecular processes, so understanding the relationship between protein structure and protein function is of utmost importance to biologists and other life scientists. Here, we study the interaction between binary protein complexes–pairs of protein structures that bind together–to better understand how these coupled proteins will function *in vivo*, as illustrated in Fig. 1. Predicting where two proteins will interface *in silico* has become an appealing method for measuring the interactions between proteins as a computational approach saves time, energy, and resources compared to traditional methods for experimentally measuring such interfaces^1^.

A key motivation for determining protein-protein interface regions is to decrease the time required to discover new drugs and to advance the study of newly designed and engineered proteins^2^. Towards this end, we set out to curate a dataset large enough and with enough features to develop computational models that can reliably predict the residues that will form the interface between two given proteins. In response to the exponential rate of progress being made in applying representation learning to biomedical data, we designed a dataset to accommodate the need for more detailed features indicative of interacting protein residues to solve this fundamental problem in structural biology, since deep learning models in computational biology often require large, feature-rich datasets for model training^3–5^.

Overall, two main encoding schemes have been proposed for protein interface prediction: modeling protein structures at the atomic level and modeling structures at the level of the residue. Modeling protein structures in terms of their atoms can yield a detailed representation of such geometries, however, accounting for each atom in a structure can quickly become computationally burdensome or infeasible for large structures. On the other hand, modeling only a structure’s residues allows one to employ their models on a more computationally succinct view of the structure, thereby reducing memory requirements for the training and inference of biomolecular machine learning models by focusing only on the alpha-carbon (C*α*) atoms of each residue. The latter scheme also enables researchers to curate robust residue-based features for a particular task, a notion of flexibility quite important to the success of prior works in protein bioinformatics^6–9^.

Nonetheless, both schemes, when adopted by a machine learning algorithm such as a neural network, require copious amounts of training examples to generalize past the training dataset. However, only a handful of extensive datasets for protein interface prediction currently exist, DIPS being the largest of such examples, and it is designed solely for modeling structures at the atomic level. If one would like to model complexes at the residue level to summarize the structural and functional properties of each residue’s atoms as additional features for training, the modest Docking Benchmark 5 (DB5) dataset is currently one of the only datasets with readily-available pairwise residue labels that meets this criterion. As such, one of the primary motivations for curating DIPS-Plus^10^ was to answer the following two questions: Must one choose between having the largest possible dataset and having enough features for their interface prediction models to generalize well? Is it possible for a single dataset to facilitate both protein-encoding schemes while maintaining its size and feature-richness?

As a follow-up to the above two questions, we constructed DIPS-Plus^10^, a feature-expanded version of DIPS, accompanied, with permission from the original authors of DIPS, by a CC-BY 4.0 license for reproducibility and extensibility. This dataset can be used with most deep learning algorithms, especially geometric learning algorithms (e.g., CNNs, GNNs), for studying protein structures, complexes, and their inter/intra-protein interactions at scale. It can also be used to test the performance of new or existing geometric learning algorithms for node classification, link prediction, intrinsically disordered interface region prediction, or similar benchmarking tasks. In the remainder of this work, we will describe how we constructed DIPS-Plus^10^ and how others can use DIPS-Plus^10^ for training new machine learning models for protein interface prediction.

## Methods

### Original collection process

The data associated with each entry in DIPS-Plus^10^ originates from the Research Collaboratory for Structural Bioinformatics (RCSB) repository for Protein Data Bank (PDB) protein complexes, where each complex was screened, inspected, and analyzed by biomedical professionals and researchers before being deposited into the RCSB PDB. To determine each protein complex’s 3D structure, X-ray diffraction, nuclear magnetic resonance (NMR), and electron microscopy (EM) are the most common experimental methods for ascertaining new complex structures. These techniques are industry standard in biomolecular research. Such protein structures in the RCSB PDB have been collected diligently over the last 50 years.

### Construction of DIPS-Plus

DIPS-Plus^10^ was developed as an extension of the original DIPS dataset^11^ by first downloading PDB file archives from the RCSB according to the original dataset’s instructions on GitHub. All downloaded archive files were then extracted using a Python extraction script. The downloaded entries were subsequently converted into a pairwise representation for protein chains within a given complex using a Python collation script, after which all pairs were redundancy-reduced using a 30% sequence identity filter to prevent data leakage between dataset partitions using a Python filtering script. New features were added to each protein complex entry using a script for collecting PSAIA-derived features and a script for organizing HHsuite-derived features. In addition to these features, features derived from DSSP and other external tools were added to each dataset entry using a dataset formatting script and a feature construction script. Lastly, DIPS-Plus’^10^ protein complexes were split into training and validation partitions using a partitioning script; were analyzed using a statistics collection script and reporting script; and had missing feature values imputed using a feature imputation script. Optionally, using a data conversion script, DIPS-Plus’^10^ protein complexes were also converted into protein graphs to train deep learning algorithms on such examples. At the end of the dataset construction process, DIPS-Plus^10^ was subsequently comprised of 42,112 complexes compared to the 42,826 complexes in the original DIPS dataset^11^ after pruning out 714 large and evolutionarily-distinct complexes for which multiple sequence alignment (MSA) generation was prohibitively time-consuming and computationally expensive.

Regarding the choice to base the proposed dataset on DIPS^11^, we made this decision for a variety of reasons. The first is that DIPS^11^ has been widely adopted by the machine learning community for evaluating the behavior of different machine learning algorithms for predicting protein-protein interactions. Therefore, extending DIPS^11^ should build on top of its recent success and contributions. Another important point of consideration was that, in effect, DIPS^11^ is one of the largest, high-quality subsets of all complexes in the Protein Data Bank (PDB). By employing carefully designed redundancy reduction filters to derive high-quality binary protein complexes, DIPS represents a desirable subset of protein complexes for training machine learning models, in that such structures are of reasonable structural quality and, importantly, are also limited in size to establish an upper bound on the computational complexity of training models on DIPS^11^. This last point allows one to quickly and effectively experiment with DIPS^11^ using different kinds of machine learning algorithms that include a variety of inductive biases.

### Assembly of new features

A novel set of statistical and geometric features were compiled for each protein complex in DIPS-Plus^10^. Each of these features was selected carefully and intentionally based on our analysis of previous, successful interface prediction models. In this section, we describe each of these new features in detail, including why we chose to include them, how we collected or generated them, and the strategy we took for normalizing the features and imputing missing feature values when they arose. These features were derived only for standard residues (e.g., amino acids) by filtering out hetero residues and waters from each PDB complex before calculating, for example, half-sphere amino acid compositions for each residue. This is, in part, to reduce the computational overhead of generating each residue’s features. More importantly, however, we chose to ignore hetero residue features in DIPS-Plus^10^ to keep it consistent with DB5 as hetero residues and waters are not present in DB5.

DIPS-Plus^10^, compared to DIPS, not only contains the original PDB features in DIPS such as amino acids’ Cartesian coordinates and their corresponding atoms’ element types but now also new residue-level features shown in Table 1 following a feature set similar to^6–8^. DIPS-Plus^10^ also replaces the residue sequence-conservation feature conventionally used for interface prediction with a novel set of emission and transition probabilities derived from HMM sequence profiles. Each HMM profile used to ascertain these residue-specific transition and emission probabilities was constructed by HHmake^12^ using MSAs that were generated after two iterations by HHblits^12^ and the Big Fantastic Database (BFD) (version: March 2019) of protein sequences^13^. Inspired by the work of Guo *et al*.^9^, HMM profiles were used to create sequence-based features in DIPS-Plus^10^, as such profiles have been shown to contain more detailed information concerning the relative frequency of each amino acid in alignment with other protein sequences compared to what has traditionally been done to generate sequence-based features for interface prediction (e.g., directly sampling MSAs to assess how conserved each residue is^12^).

**Table 1:** Residue features included in DIPS-Plus^10^.

New Features (1)	New Features (2)
Secondary Structure	Coordinate Number
Relative Solvent Accessibility	Profile HMM Features
Residue Depth	Amide Normal Vector
Protrusion Index	Intrinsic Disorder Region
Half-Sphere Amino Acid Composition	

Furthermore, in DIPS-Plus’^10^ GitHub repository, users are provided with a Jupyter notebook describing how they can generate each of the proposed protein features for a protein sequence and structure within a given PDB file. In addition, this notebook also shows users how to post-process their generated protein features into a graph representation amenable to representation learning with popular graph neural network libraries such as the Deep Graph Library (DGL)^14^. To train new deep learning models using such features, users are also provided with a Jupyter notebook that allows one to train new models using the proposed feature set. Lastly, using DIPS-Plus’ feature insertion script, users are able to add to the dataset any residue-level feature sets provided by the Graphein library^15^, of which there are currently several dozens.

#### Secondary structure

Secondary structure (SS) annotations were included in DIPS-Plus^10^ as a categorical variable that describes the type of local, three-dimensional structural segment in which a residue can be found. This feature has been shown to correlate with the presence or absence of protein-protein interfaces^16^. In addition, the secondary structures of residues are informative of the physical interactions between main-chain and side-chain groups^17^. This is one of the primary motivations for including them as a residue feature in DIPS-Plus^10^. As such, we hypothesized that adding secondary structure as a feature for interface prediction models could prove beneficial to model performance as it would allow them to more readily discover interactions between structures’ main-chain and side-chain groups. Each residue’s SS value was generated using version 3.0.0 of the Database of Secondary Structure Assignments for Proteins (DSSP)^18^, a well-known and frequently-used software package in the bioinformatics community. Here, the BioPython interface to DSSP (version 1.78)^19^ was used to retrieve the DSSP results for each residue. Each residue is assigned one of eight possible SS values, ‘H’, ‘B’, ‘E’, ‘G’, ‘I’, ‘T’, ‘S’, or ‘-’, with the symbol ‘-’ signifying the default value for unknown or missing SS values. Since this categorical feature is naturally one-hot encoded, it did not need to be normalized numerically.

#### Relative solvent accessibility

Each residue can behave differently when interacting with water. Solvent accessibility is a scalar (i.e., type 0) feature that quantifies a residue’s accessible surface area, the area of a residue’s atoms that can be touched by water. Polar residues typically have larger accessible surface areas, while hydrophobic residues tend to have a smaller accessible surface area. It has been observed that hydrophobic residues tend to appear in protein interfaces more often than polar residues^20^. Including solvent accessibility as a residue-level feature, then, may provide models with additional information regarding how likely a residue is to interact with another inter-protein residue.

Relative solvent accessibility (RSA) is a simple modification of solvent accessibility that normalizes each residue’s solvent accessibility by an experimentally-determined normalization constant specific to each residue. These normalization constants are designed to help more closely correlate generated RSA values with their residues’ true solvent accessibility^21^. Subsequently, each residue in each DIPS-Plus^10^ protein complex was assigned an RSA value using BioPython’s DSSP interface. Specifically, the RSA values returned from BioPython were pre-normalized according to the constants described in^21^ and capped to an upper limit of 1.0. Missing RSA values were denoted by the NaN constant from NumPy^22^, a popular scientific computing library for Python. Thus, users of DIPS-Plus^10^ may easily impute missing feature values for each feature type; scripts with default parameters to do so were correspondingly compiled with the source code for DIPS-Plus^10^. By default, NaN values within DIPS-Plus^10^ for numeric features such as RSA were imputed using the feature’s columnwise median value.

#### Residue depth

Residue depth (RD) is a scalar measure of the average distance of the atoms of a residue from its solvent-accessible surface. Afsar *et al*.^6^ have found that for interface prediction this feature is complementary to each residue’s RSA value. Hence, this feature holds predictive value for determining interacting protein residues as it can be viewed as a description of how “buried” each residue is. As such, BioPython and version 2.6.1 of MSMS^23^ were used to generate each residue’s depth for DIPS-Plus^10^, where the default quantity for a missing RD value is NaN. To make all RD values fall within the range [0, 1], structure-specific min-max normalization of each structure’s non-NaN RD values was performed using scikit-learn^24^. That is, for each structure, the structure’s RD values were normalized using the expression

1 $${X}_{normalized}=\frac{X-b}{t-b}\times \left(u-l\right)+l,$$

where $X\in {{\mathbb{R}}}^{n\times d}$ represents a specific feature of a protein complex (e.g., RD values); *n* is the number of residues in a protein complex; *d* is the number of dimensions in the given protein feature; *l* and *u*, respectively, represent the lower and upper scalar bounds to which to normalize the feature values in *X*; and *b* and *t*, respectively, are the original residue-wise minimum and maximum feature values for a given feature *X*. Here, the values *l* = 0 and *u* = 1 were used.

#### Protrusion index

A residue’s protrusion index (PI) is defined using its non-hydrogen atoms. It is a measure of the proportion of a 10 Å sphere centered around the residue’s non-hydrogen atoms that is not occupied by any atoms. By computing protrusion this way, one ends up with a 1 × 6 feature vector that describes the following six properties of a residue’s protrusion: average and standard deviation of protrusion, minimum and maximum protrusion, and average and standard deviation of the protrusion of the residue’s non-hydrogen atoms facing its side chain. Version 1.0 of PSAIA^25^ was used to calculate the PIs for each DIPS-Plus^10^ structure’s residues collectively. That is, each structure had its residues’ PSAIA values packaged in a single .tbl file. Missing PIs defaulted to a 1 × 6 vector consisting entirely of NaNs. Each PI entry was min-max normalized columnwise to get six updated PI values, similar to how RD values were normalized in a structure-specific manner.

#### Half-sphere amino acid composition

Half-sphere amino acid compositions (HSAACs) are comprised of two 1 × 21 unit-normalized vectors concatenated together to get a single 1 × 42 feature vector for each residue. The first vector, termed the upward composition (UC), reflects the number of times a particular amino acid appears along the residue’s side chain, while the second, the downward composition (DC), describes the same measurement in the opposite direction, with the 21st vector entry for each residue corresponding to the unknown or unmappable residue, ‘-’. Knowing the composition of amino acids along and away from a residue’s side chain, for all residues in a structure, is another feature that has been shown to offer crucial predictive value to machine learning models for interface prediction as it can describe physiochemical and geometric patterns in such regions^26^. These UC and DC vectors can also vary widely for residues, suggesting an alternative way of assessing residue accessibility^6,8^. In DIPS-Plus^10^, missing HSAACs were imputed using a 1 × 42 vector consisting entirely of NaNs. Furthermore, since both the UC and DC vectors for each residue were unit normalized before concatenating them together, after concatenation all columnwise HSAAC values for a structure still inclusively fell between 0 and 1.

#### Coordinate number

A residue’s coordinate number (CN) is conveniently determined alongside the calculation of its HSAAC. It denotes how many other residues to which the given residue was found to be significant. Significance, in this context, was defined in the same way as^6^. That is, the significance score for two residues was defined as

2 $$s={e}^{\frac{-{d}^{2}}{2s{t}^{2}}},$$

where *d* is the minimum distance between any of their atoms and *st* is a given significance threshold which, in the context of DIPS-Plus^10^, defaulted to the constant 1*e*^−3^. Then, if two residues’ significance score rose above *st*, they were considered significant. As per the standard convention in DIPS-Plus^10^, the default value for missing CNs was NaN, and the CN for each structure’s residues was min-max normalized.

#### Profile HMM features

MSAs can carry rich evolutionary information regarding how each residue in a structure is related to all other residues, and sequence profile HMMs have increasingly found use in representing MSAs’ evolutionary information in a concise manner^12,27^. In previous works on PIP, knowing the conservation of a residue has been beneficial in predicting whether the residue is likely to be found in an interface^6–8^, and profile HMMs capture this sequence conservation information in a novel way using MSAs. As such, to gather sequence profile features for DIPS-Plus^10^, profile HMMs were derived for each structure’s residues using HH-suite3 by first generating MSAs using HHblits followed by taking the output of HHblits to create profile HMMs using HHmake. From these profile HMMs, each DIPS-Plus^10^ structure’s residue-wise emission and transition profiles could then be calculated. A residue’s emission profile, represented as a 1 × 20 feature vector of probability values, subsequently illustrated how likely the residue is across its evolutionary history to emit one of the 20 possible amino acid symbols. Similarly, each residue’s transition profile, a 1 × 7 probability feature vector, then depicted how likely the residue is to transition into one of the seven possible HMM states.

To derive each structure’s emission and transition probabilities, for a residue *i* and a standard amino acid *k* the profile HMM entry (*i, k*) (i.e., the corresponding frequency) was extracted and the frequency (Freq) was converted into a probability value with the equation

3 $${p}_{ik}={2}^{-\frac{{{\rm{Freq}}}_{ik}}{m}},$$

where *m* is the number of MSAs used to generate each profile HMM (*m* = 1,000 by default).

After doing so, a 1 × 27 vector of probability values was obtained for each residue. Similar to other features in DIPS-Plus^10^, missing emission and transition probabilities for a single residue were set to a 1 × 27 vector comprised solely of NaNs. Moreover, since each residue was assigned a probability vector as its sequence features, we did not need to normalize these sequence feature vectors columnwise. We chose to leave out three profile HMM values for each residue representing the diversity of the alignment concerning HHmake’s generation of profile HMMs from HHblits’ generated MSAs for a given structure. Since we did not see any predictive value in including these as residue features, we left them out of both DIPS-Plus^10^ and DB5-Plus^10^. Additionally, for users’ convenience, we also collected for each chain in DIPS-Plus^10^ MSAs that were generated using Jackhmmer^28^ and AlphaFold’s small version of the BFD^3^ as an alternative source of sequence-based data, and we have stored such alignments in DIPS-Plus’ supplementary Zenodo data repository^29^.

#### Amide normal vector

Each residue’s amide plane has a normal vector (NV) that can be derived using the expression

4 $$NV=\left({\overrightarrow{x}}_{C\alpha }-{\overrightarrow{x}}_{C\beta }\right)\times \left({\overrightarrow{x}}_{C\beta }-{\overrightarrow{x}}_{N}\right),$$

where ${\overrightarrow{x}}_{C\alpha }$, ${\overrightarrow{x}}_{C\beta }$, and ${\overrightarrow{x}}_{N}$ are the Cartesian coordinates of a residue’s *Cα*, *Cβ*, and nitrogen atoms, respectively, and × represents the cross product of two vectors.

As shown in Eq. 4, if users opted to encode the complexes in DIPS-Plus^10^ as pairs of graphs, such NVs can then be used to define rich edge features such as the angle between the amide plane NVs for two residues^7^. Similar to how other missing feature vectors were imputed, the default value for an underivable NV (e.g., for Glycine residues that do not have a beta-carbon atom) was a 1 × 3 vector consisting of NaNs. Further, since these vectors represent residues’ amide plane NVs, we left them unnormalized for, at users’ discretion, additional postprocessing (e.g., custom normalization) of these NVs.

#### Intrinsically disordered regions

To allow one to study or predict disordered protein interface regions more readily, the residues in each complex in DIPS-Plus^10^ were annotated according to whether a given residue probabilistically resides in an intrinsically disordered region (IDR). Such annotations were generated using flDPnn^30^, a state-of-the-art IDR prediction method. Each annotation is provided as both a scalar *disorder propensity* value between 0 and 1 as well as a binary classification (i.e., integer) value of 0 or 1, with 1 indicating a given residue likely resides in an IDR and 0 otherwise. Such annotations are stored in the dataset’s supplementary Zenodo data repository^29^.

### Preprocessing, cleaning, and labeling

Important to note is that all nine of the residue-level features added in DIPS-Plus^10^ were missing values for at least one residue. This is because not all residues had, for example, DSSP-derivable secondary structure (SS) values^18^ or profile hidden Markov models (HMMs) that are derivable by HH-suite3^12^, the software package used to generate multiple sequence alignments (MSAs) and subsequent MSA-based features. A similar situation occurred for the seven other residue features. That is, not all residues had derivable features for a specific feature column, governed either by DIPS-Plus’^10^ feature parsers or by the external feature parsers used in constructing DIPS-Plus^10^. Missing feature values were denoted for all features as NumPy’s NaN constant, except for residues’ SS value, in which case ‘-’ was used as the default missing feature value^22^. In the case of missing features, NumPy’s NaN constant was substituted for the missing feature value. Also provided with DIPS-Plus^10^ were postprocessing scripts with which users may perform imputation of missing feature values (e.g., replacing a column’s missing values with the column’s mean, median, minimum, or maximum value or with a constant such as zero) depending on the type of the missing feature (i.e., categorical or numeric).

Also important to note is that the version of each DIPS-Plus^10^ protein complex before any postprocessing was performed was saved separately in DIPS-Plus’^10^ dedicated Zenodo data repository^10^. That is, each pruned pair from DIPS was stored in the data repository before the addition of any DIPS-Plus^10^ features. Subsequently, the raw complexes from which DIPS complexes were derived can be retrieved from the RCSB PDB individually or in batch using FTP or similar file-transfer protocols (from the PDB).

### Novel use cases for DIPS-Plus

The original DIPS dataset, being a carefully curated PDB subset, contains almost 200× more protein complexes than the modest 230 complexes in DB5, which is still considered to be a gold standard of protein-protein interaction datasets. Other protein binding datasets such as PDBBind^31^ (containing 5,341 protein-protein complexes) and that which was used in the development of MaSIF^32^ (containing roughly 12,000 protein-protein complexes in total) have previously been curated for machine learning of protein complexes. However, to the best of our knowledge, DIPS-Plus^10^ now serves as the single largest database of PDB protein-protein complexes incorporating novel features such as profile HMM-derived sequence conservation and half-sphere amino acid compositions shown to be indicative of residue-residue interactions, as described in our technical validation study. Our data pipeline can be used to extend DIPS-Plus^10^ to include any new complexes in PDBBind or MaSIF.

## Data Records

As contained in the dataset’s primary Zenodo data repository^10^ and referenced in its supplementary Zenodo data repository^29^, DIPS-Plus’^10^ primary data records are comprised of binary protein complexes (i.e., bound ligand and receptor protein structures) extracted from the RCSB PDB^33^. Both protein structures in the complex are differentiable in that they are stored in their own Pandas DataFrame objects^34^. Each structure’s DataFrame contains information concerning the atoms of each residue in the structure such as their Cartesian coordinates and element type. For the alpha-carbon atoms of each residue (typically the most representative atom of a residue), each structure’s DataFrame also contains residue-level features like a measure of amino acid protrusion and solvent accessibility.

Each data record, consisting of a pair of DataFrames containing a series of alpha-carbon (CA) atoms and non-CA atoms with residue and atom-level features, respectively, is stored in both a Python dill file and a Hierarchical Data Format (HDF) file for data compression and convenient file loading^35^. These pairwise DataFrames subsequently contain a combination of numeric, categorical, and vector-like features describing each atom. In total, there are 42,112 data records for binary protein complexes in DIPS-Plus^10^ after completing all data pruning.

The data records contain the labels of which pairs of CA atoms from opposite structures are within 6 Å of one another (i.e., positives), implying an interaction between the two residues, along with an equally-sized list of randomly-sampled non-interacting residue pairs (i.e., negatives). For example, if a complex in DIPS-Plus^10^ contains 100 interacting residue pairs (i.e., positive instances), there will also be 100 randomly-sampled non-interacting residue pairs included in the complex’s dill file for optional downsampling of the negative class during the training of machine learning models.

**Fig. 2:**
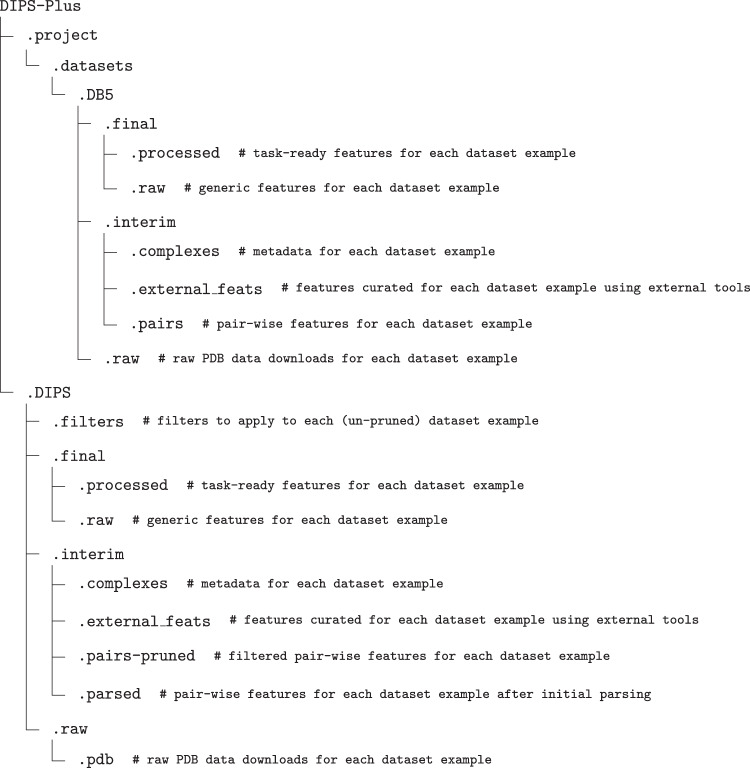
An overview of the directory structure of our proposed datasets.

As shown in the directory structure diagram in Fig. 2, the relationships between individual data records (i.e., protein complexes) are made explicit by the directory and file naming convention we adopt for DIPS-Plus^10^. Complexes’ DataFrame files are grouped into directories by shared second and third characters of their PDB identifier codes (e.g., 1x9e.pdb1_0.dill and 4x9e.pdb1_5.dill reside in the same directory project/datasets/DIPS/final/raw/x9/).

Since DIPS-Plus^10^ is relatively large (i.e., contains more than 10,000 complexes), we provide a randomly-sampled 80%-20% dataset split for training and validation data, respectively, in two different forms of text file pairs located in the directory project/datasets/DIPS/final/raw/: pairs-postprocessed-train.txt and pairs-postprocessed-val.txt corresponding to the dataset’s default structure-based splits (as discussed in the following Technical Validation section), and pairs-postprocessed-train-before-structure-based-filtering.txt and pairs-postprocessed-val-before-structure-based-filtering.txt corresponding to the dataset’s original 30% sequence identity splits (also discussed in the following Technical Validation section). The file pairs-postprocessed.txt is a master list of all complex file names from which we derived pairs-postprocessed-train.txt and pairs-postprocessed-val.txt for cross-validation. It contains the file names of 42,112 complex DataFrames, filtered down from the original 42,826 complexes in DIPS-Plus^10^ to complexes having no more than 17,500 CA and non-CA atoms, to match the maximum possible number of atoms in DB5-Plus structures and to create an upper-bound on the computational complexity of learning algorithms trained on DIPS-Plus^10^. However, we have also included the scripts necessary to conveniently regenerate pairs-postprocessed.txt with a modified or removed atom-count filtering criterion and with different cross-validation ratios.

Note that, to generate each data record, DIPS-Plus^10^ relies on feature generation using external tools such as DSSP and PSAIA. However, in our Zenodo data repository for DIPS-Plus^10^, we provide either a copy of the external features generated using these tools or the exact version of the tool with which we generated features (e.g., version 3.0.0 of DSSP for generating SS values using version 1.78 of BioPython). Each of these externally-generated features is represented by the external_feats directory in Fig. 2. The most time-consuming and computationally-expensive features to generate, profile HMMs and protrusion indices, are included in our Zenodo repository for users’ convenience. We also provide the final, postprocessed version of each DIPS-Plus^10^ complex in our Zenodo data bank, shown as the final/raw directory in Fig. 2.

## Technical Validation

### Data quality

Regarding the quality and validity of the complexes in DIPS-Plus^10^, we employed a similar pruning methodology as^36^ to ensure data integrity. DIPS-Plus, along with the works of others^31,32,37^, derives its complexes from the PDB which conducts statistical quality summaries in its structure deposition processes and post-deposition analyses^38^. Nonetheless, recent studies on the PDB have discovered that the quality of its structures can, in some cases, vary considerably between structures^39^. As such, in selecting complexes to include in DIPS-Plus^10^, we performed extensive filtering and processing after obtaining the initial batch of 180,000 protein structures available in the PDB. Such filtering included (1) selecting only structures that contain at least two chains (i.e., complex structures); (2) removing PDB complexes containing a protein chain with more than 30% sequence identity with any protein chain in DB5-Plus per^40,41^; (3) selecting complexes with an X-ray crystallography or cryo-electron microscopy resolution greater than 3.5 Å (i.e., a standard threshold in the field); (4) choosing complexes containing protein chains with more than 50 amino acids (i.e., residues); (5) selecting for complexes with at least 500 Å^2^ of buried surface area; and (6) picking only the first model for a given complex. Lastly, for each of the complex structures we retained after such filtering steps, we then found all unique pairs of chains in each complex and represented them as individual protein complexes (i.e., as binary complexes) for training and cross-validation of machine learning models. The motivation for the second filtering step was to ensure that we did not allow training datasets built from DIPS-Plus^10^ to bias the DB5-Plus test results of models trained on DIPS-Plus^10^, with the remaining steps carried out to follow standard practices in the field of protein bioinformatics.

In addition to offering a standardized sequence-based split of the dataset’s complexes, the dataset also provides a default structure-based split of these complexes. In particular, DIPS-Plus offers a standardized train-validation partitioning of the dataset’s complexes using FoldSeek^42^, such that there are no complexes (i.e., chains) within the dataset’s original sequence-based training or validation splits that are structurally similar to any chain within the DB5 dataset’s test split. To create such structure-based splits, FoldSeek was run using DIPS-Plus’ data partitioning script in exhaustive search mode between all chains in the training and validation dataset and the chains in the DB5 test dataset. In this context, a chain was considered structurally similar to another chain if FoldSeek assigned a 50% or greater probability to the two chains belonging to the same SCOPe superfamily, while permitting E-values up to 0.1. Note that the default E-value upper limit for FoldSeek is 0.001, which means that the use of an increased upper limit on E-values in DIPS-Plus’ FoldSeek searches modified the similarity searches for all chain pairs to report more distant potential homologs compared to FoldSeek’s default search settings. After performing this exhaustive search, FoldSeek removed 3,727 of the 33,159 original sequence-filtered chain pairs within the DIPS-Plus training split, resulting in 29,432 chain pairs remaining for training. Similarly, FoldSeek removed 890 of the 8,290 original sequence-filtered chain pairs within the DIPS-Plus validation split, resulting in 7,400 chain pairs remaining for validation. Nonetheless, if one desires to customize FoldSeek’s data splitting behavior further, one can use DIPS-Plus’ data partitioning script to do so.

To inspect DIPS-Plus’ training and validation complexes further, we additionally provide within DIPS-Plus’ GitHub repository a complex experiment analysis script that uses the Graphein library’s latest PDBManager utility^15^ to collect and organize detailed metadata associated with the protein chains in DIPS-Plus. This metadata includes not only each chain’s experiment type and resolution for structure determination but also its PDB name (e.g., “Adipocyte Lipid Binding Protein”), sequence, the PDB identifiers of ligands originally in complex with the chain, the source of the chain (e.g., “homo sapiens”), and its PDB deposition date. We provide this metadata for DIPS-Plus within the latest version of its supplementary Zenodo data record^29^.

**Fig. 3:**
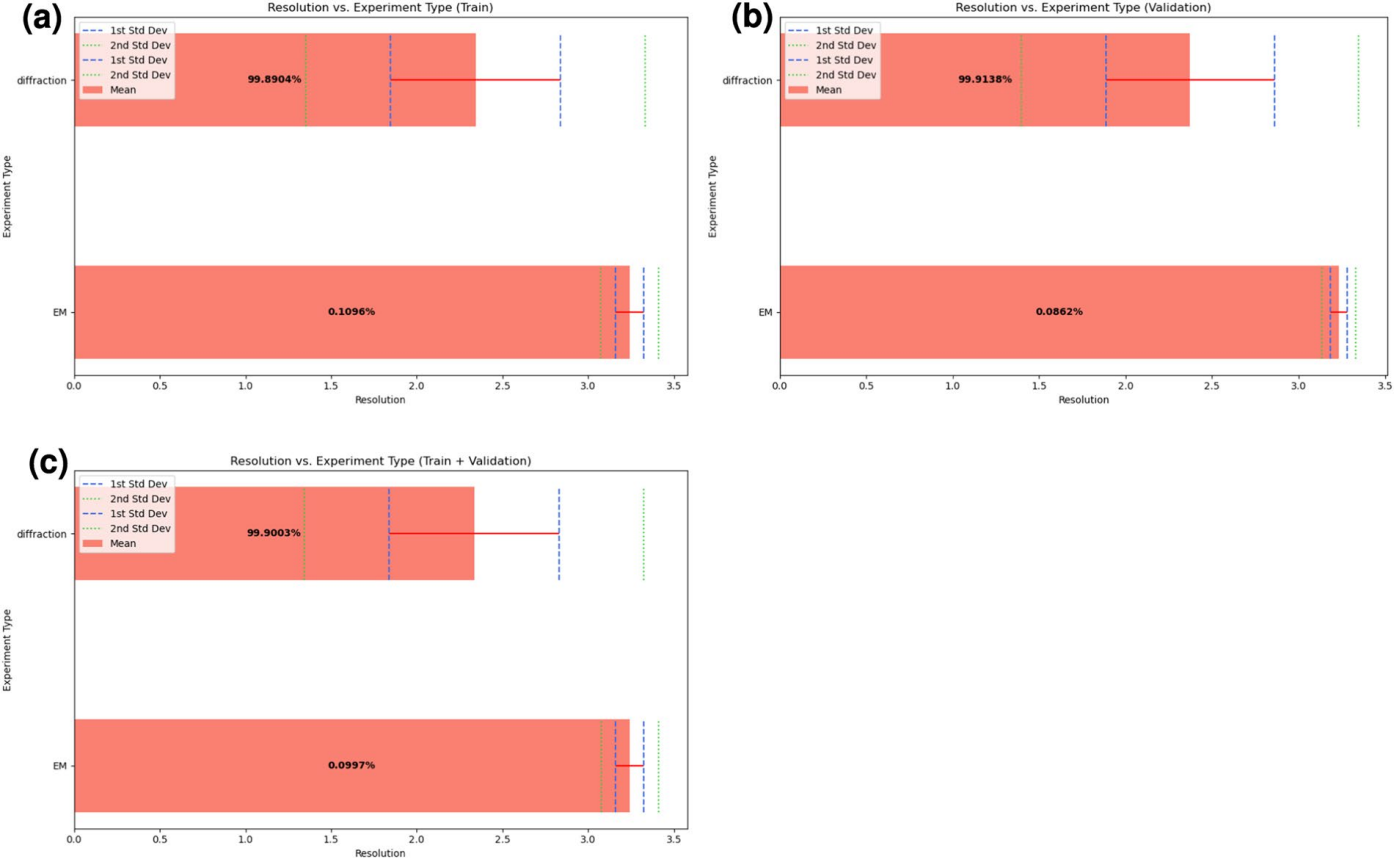
Displays the distribution of experiment types and resolutions across DIPS-Plus’ training and validation examples.

In particular, Fig. 3 visualizes the distribution of each DIPS-Plus chain’s experiment resolution values along with its experiment resolution method. As Fig. 3 shows, the training and validation DIPS-Plus data splits have very similar distributions in terms of experiment resolutions achieved and experiment methods used, with both splits having a similar spread of resolution values between 1.4 and 3.5 Angstrom. Across both splits, the average resolution of diffraction-derived chains is approximately 2.3 Angstrom, and the average resolution of electron microscopy (EM)-derived chains is approximately 3.2 Angstrom. Given that diffraction-derived chains comprise approximately 99% of the chains in DIPS-Plus, this analysis suggests that, on average, the quality of the interfaces present within DIPS-Plus is reasonable and well-balanced between both its training and validation splits, with each split containing mostly lower-resolution diffraction-derived chains but also containing some intermediate-resolution EM-derived chains.

**Fig. 4:**
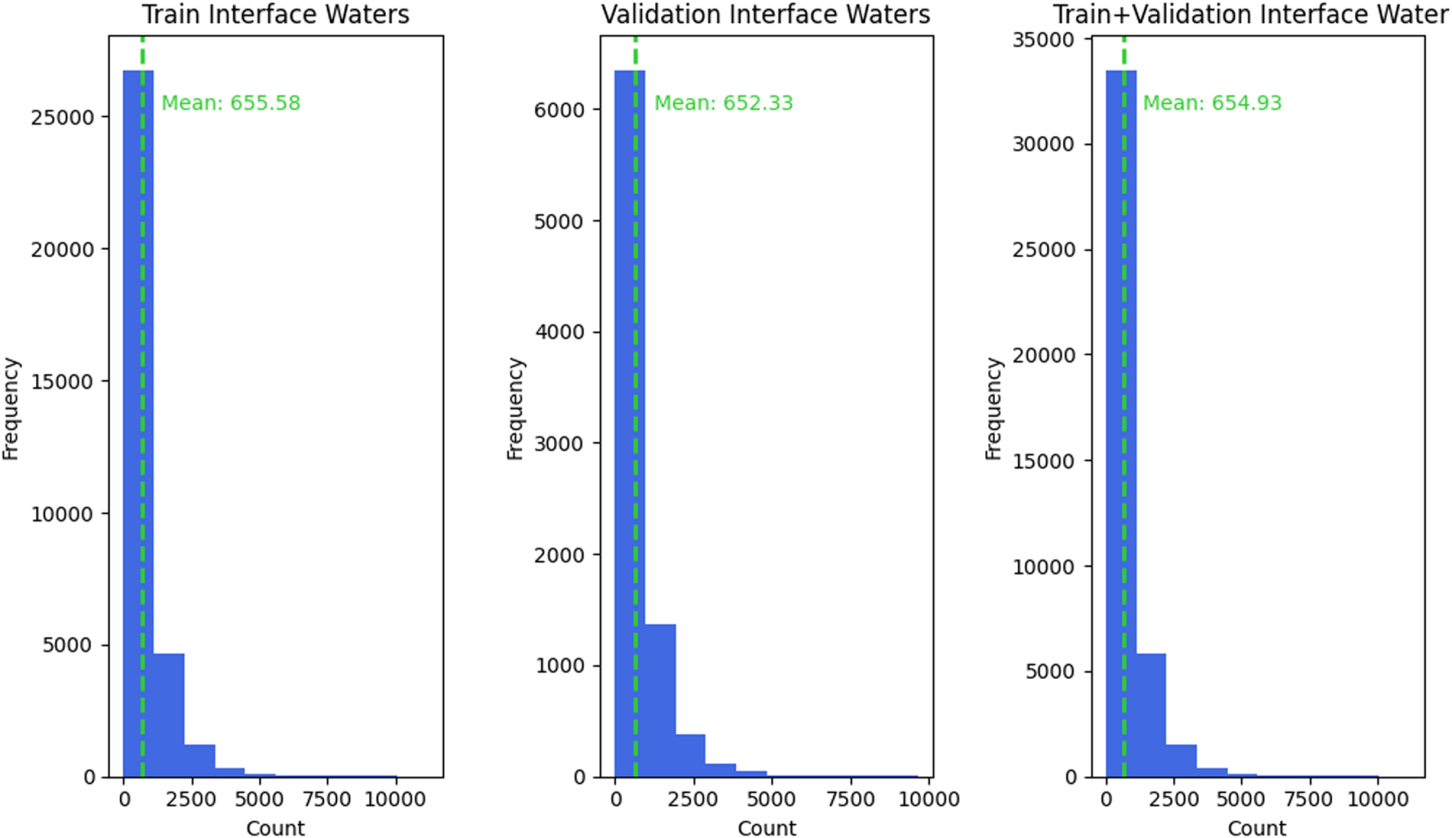
An investigation of the distribution of the number of interface water atoms interacting with interface residues across the complexes within DIPS-Plus.

Another perspective through which to validate the dataset’s quality is through an analysis of its original number of water atoms present in each protein-protein interface, prior to removal of water atoms to closely mirror the DB5 dataset. Towards this end, included within DIPS-Plus’ GitHub repository is a complex interface water analysis script that allows users to plot the distribution of how many atoms associated with a water molecule (e.g., an ‘HOH’ residue) are located within 10 Angstrom of any residue residing in given protein-protein interface for each complex. An analysis performed using this script, as shown in Fig. 4, demonstrates that around 656, 652, and 655 water atoms, on average, are present in each protein-protein interface within DIPS-Plus’ training, validation, and training with validation complexes, respectively. This indicates, in mimicking the lack of waters present in the DB5 dataset, that certain amino acid-water interactions within each DIPS-Plus complex may have to be inferred by machine learning methods trained on DIPS-Plus (or on DB5, consequently) for downstream protein interface prediction.

However, the discussion in the following Usage Notes section highlights, using the new feature pipeline introduced with DIPS-Plus’ source code, that users of DIPS-Plus are able to add to the dataset a rich new set of descriptors, such as hydrophobicity embeddings, that can help characterize the various amino acid-water interactions that can occur at protein-protein interfaces. Since this feature pipeline allows users to featurize protein graphs constructed from PDB file inputs at the level of amino acids or all atoms, users now can customize how they would like to featurize each protein-protein complex in DIPS-Plus to capture different characteristics of amino acid-water interactions from the perspective of either amino acid sequences or protein tertiary structures.

**Fig. 5:**
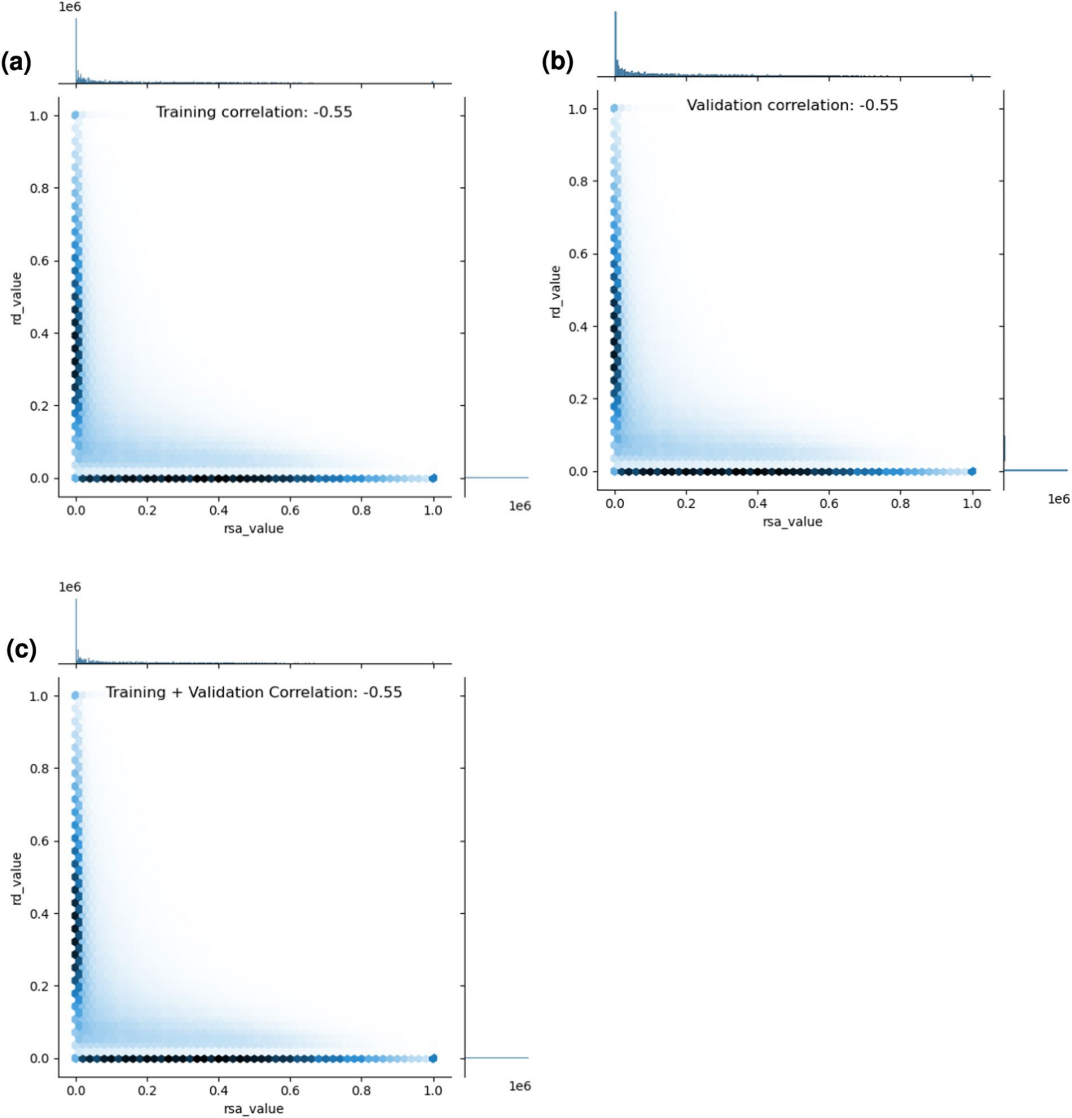
An investigation of the correlation between per-residue relative solvent accessibility (RSA) and residue depth (RD) values across DIPS-Plus’ training and validation examples.

To further investigate data quality, we also looked at whether certain features in the dataset are highly correlated with one another, implying feature redundancy. In particular, Fig. 5 quantitatively and qualitatively shows the relationship between relative solvent accessibility (RSA) and residue depth (RD) within DIPS-Plus. As shown in Fig. 5, the two features are moderately correlated with each other, yet not entirely, with a Pearson’s correlation of −0.55 across the training and validation splits. As Fig. 4 further illustrates, there are regions in the two features’ distributions where they do not strongly overlap (e.g., between 0.01 and 0.2 for RD values), suggesting that there is still information to glean from processing these two features as separate values.

**Fig. 6:**
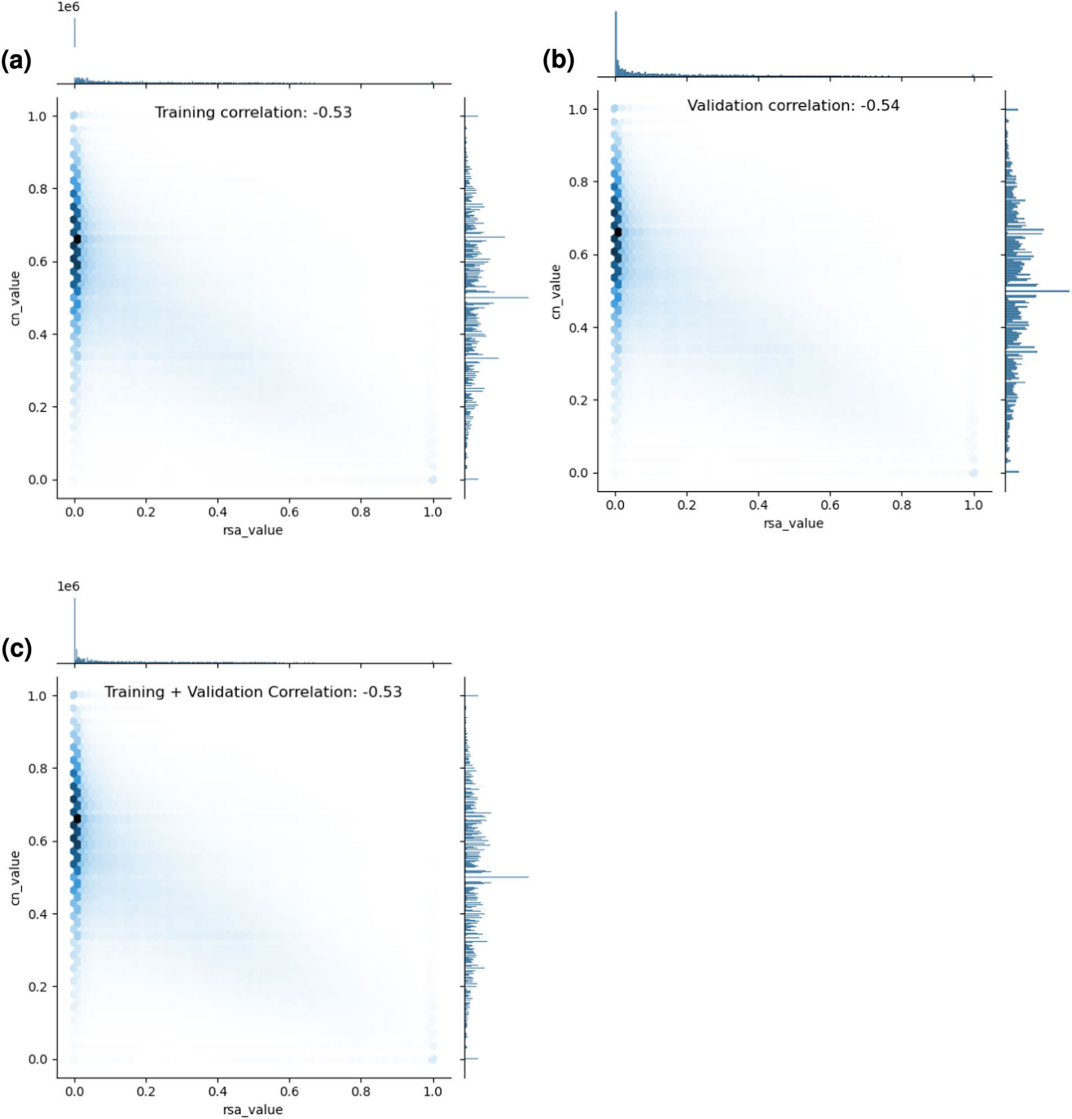
An investigation of the correlation between per-residue relative solvent accessibility (RSA) and coordinate number (CN) values across DIPS-Plus’ training and validation examples.

**Fig. 7:**
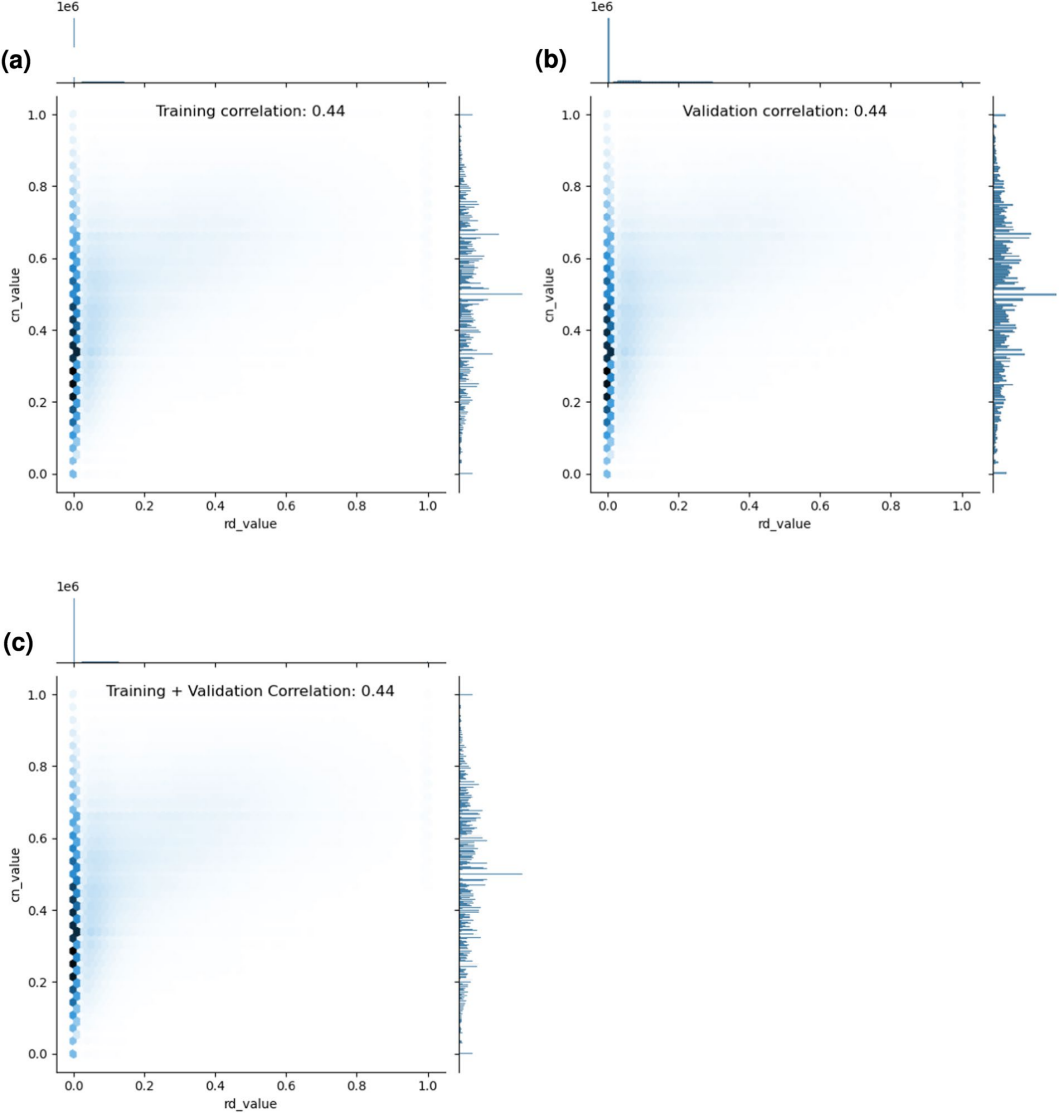
An investigation of the correlation between per-residue residue depth (RD) and coordinate number (CN) values across DIPS-Plus’ training and validation examples.

Similarly, we also investigated the correlation between RSA values and each residue’s coordinate number (CN) values as well as RD values and each residue’s CN values, to span each of the dataset’s single-valued scalar features. The results of these two additional investigations are shown below in Figs. 6, 7, respectively. In short, we find that RSA values and CN values are also moderately correlated with each other, yet not fully correlated, and similarly, yet less so, for RD values and CN values. These results, along with a qualitative analysis of each feature’s distribution, suggest that there is still predictive value to be found in treating each of these features as a separate column within the dataset. Note that users of the dataset may use its provided complex feature correlation analysis script to run additional or customized feature correlation analysis studies as desired.

### Computational benchmark results

To measure the effect that DIPS-Plus^10^ has on the performance of existing machine learning methods for PIP, we trained one of the latest SOTA methods, NeiA, for 10 epochs on our standardized 80%-20% cross-validation split of DIPS-Plus’^10^ complexes to observe NeiA’s behavior on DB5-Plus’s test complexes thereafter. In line with previous PIP studies, we ran this experiment three times, each with a random seed and a single GNN layer, for a fair comparison of the experiment’s mean (before ±) and standard deviation (after ±) in terms of MedAUROC. Our results from this experiment are shown in the last row of Table 2. For the experiment, we used the following architecture and hyperparameters: (1) 1 NeiA GNN layer; (2) 3 residual CNN blocks, each employing a 2D convolution module, ReLU activation function, another 2D convolution module, followed by adding the block input’s identity map back to the output of the block (following a design similar to that of^8^); (3) an intermediate channel dimensionality of 212 for each residual CNN block; (4) a learning rate of 1e-5; (5) a batch size of 32; (6) a weight decay of 1e-7; and (7) a dropout (forget) probability of 0.3.

**Table 2:** Effect of training on DIPS-Plus^10^ and DB5-Plus^10^ for NeiA, a state-of-the-art PIP algorithm.

Method	MedAUROC
NGF (DB5)^43^	0.865 ± 0.007
DTNN (DB5)^44^	0.867 ± 0.007
Node and Edge Average (DB5)^7^	0.876 ± 0.005
BIPSPI (DB5)^45^	0.878 ± 0.003
SASNet* (DIPS)^36^	0.885 ± 0.009
NeiA + HOPI (DB5)^8^	0.902 ± 0.012
NeiWA + HOPI (DB5)^8^	0.908 ± 0.019
**NeiA** + **HOPI (DB5-Plus)**^10^	0.9415 ± 0.000
**NeiA** + **HOPI (DIPS-Plus)**^10^	**0.9473** ± **0.001**

All baseline results on the DB5 test complexes in Table 2 (i.e., complexes comprised of original DB5 residue features)^7,8,43–45^ are taken from^8^, except for SASNet’s results from training on the original DIPS dataset. These results are denoted by an asterisk in Table 2 to indicate that they were instead taken from^36^. The best performance is in bold. Note that, for fair comparisons with prior methods for protein interface prediction, the computational benchmark results described in this section are all based on a 30% sequence identity split of each baseline method’s training data with respect to the DB5 test dataset’s chains.

In Table 2, we see that a simple substitution of training and validation datasets enhances the MedAUROC of NeiA when adopting its accompanying high-order pairwise interaction (HOPI) module for learning inter-protein residue-residue interactions. For reference, to the best of our knowledge, the best performance of a machine learning model trained for PIP on only the atom-level features of protein complexes is SASNet’s MedAUROC of **0.885** averaged over three separate runs^36^. Such insights suggest the utility and immediate advantage of using DIPS-Plus’^10^ residue feature set for PIP over the original DIPS’ atom-level feature set. Additionally, we deduce from Table 2 that the performance of previous methods for PIP is likely limited by the availability of residue-encoded complexes for training as all but one method^36^ used DB5’s 230 total complexes for training, validation, as well as testing. This hypothesis is supported by the results in Table 2 in that NeiA + HOPI trained and tested on DB5-Plus achieves a MedAUROC of 0.9415, whereas NeiA + HOPI trained on DIPS-Plus^10^ and tested on DB5-Plus achieves a new SOTA MedAUROC of 0.9473, demonstrating the importance of increasing the amount of residue-encoded complex data for PIP.

## Usage Notes

The standardized task for which DIPS-Plus^10^ is designed is the pairwise prediction of all possible interactions between inter-protein residues (e.g., *M* × *N* possible interactions where *M* and *N* are the numbers of residues in a complex’s first and second structure, respectively)^8^. One of the most common metrics used to score computational methods for PIP is the median area under the receiver operating characteristic curve (MedAUROC) to prevent test results for extraordinarily large complexes from having a disproportionate effect on the algorithm’s overall test MedAUROC^6–8,36^. To facilitate convenient training of future methods trained on DIPS-Plus^10^, DIPS-Plus^10^ includes a standardized 80%-20% cross-validation split of its complexes’ file names. For these splits, we *a priori* filtered out 663 complexes containing more than 1,000 residues to mirror DB5 in establishing an upper bound on the computational complexity of algorithms trained on the dataset. As is standard for interface prediction^6–8,36^, we defined the labels in DIPS-Plus^10^ to be the IDs (i.e., Pandas DataFrame row IDs^34^) of inter-protein residue pairs that, in the complex’s bound state, can be found within 6 Å of one another, using each residue’s non-hydrogen atoms for performing distance measurements (since hydrogen atoms are often not present in experimentally-determined structures).

Similar to^36^, in the version of DB5 we updated with new features from DIPS-Plus^10^ (i.e., DB5-Plus), we recorded the file names of the complexes added between versions 4 and 5 of Docking Benchmark as the final test dataset for users’ convenience. The rationale behind this choice of test dataset is given by the following points: (1) The task of interface prediction is to predict how two *unbound* (i.e., not necessarily conformal) proteins will bind together by predicting which pairs of residues from each complex will interact with one another upon binding; (2) DIPS-Plus^10^ consists solely of *bound* protein complexes (i.e., those already conformed to one another), so we must test on a dataset consisting of *unbound* complexes after training to verify the effectiveness of the method for PIP; (3) Each of DB5-Plus’ *unbound* test complexes are of varying interaction types and difficulties for prediction (e.g., antibody-antigen, enzyme substrate), simulating how future unseen proteins (e.g., those discovered at the outset of an epidemic) might be presented to the model following its training; (4) DB5’s test complexes (i.e., those added between DB4 and DB5) represent a time-based data split also used for evaluation in^7,8,36^, so for fair comparison with previous SOTA methods we chose the same complexes for testing.

In general, DIPS-Plus^10^ can be used with most machine learning algorithms, especially geometric deep learning algorithms, for studying protein structures, complexes, and their inter/intra-protein interactions at scale. This dataset can also be used to test the performance of new or existing geometric learning algorithms for node classification, link prediction, intrinsic disorder prediction, or similar benchmarking tasks. Important to note is that this data is collected solely in the domain of proteomics, so systems trained on it may or may not generalize to other tasks in the life sciences.

To allow users of DIPS-Plus to add new features to the dataset as desired, included within DIPS-Plus’ GitHub repository is a feature insertion script that one can use to include new features of arbitrary data types. For example, one may use the script to insert new scalar-valued features for each residue (or atom) by inserting the new features within each example’s ‘sequences’ object (i.e., dictionary) mapping string keys to arbitrary data types such as a list of scalar values. If one would like to assign new vector-valued features to each dataset example, one can instead insert use this feature insertion script to insert such vector feature values as a column vector associated with each row in an example’s ‘df0’ or ‘df1’ Pandas DataFrame. Here, ‘df0’ represents the first protein partner in a protein-protein interface, and likewise ‘df1’ represents the second partner in the interface. Since the Pandas Python library^34^ allows users to store vector values as column values, this script is able to integrate both new scalar and vector-valued features within each dataset example.

To show users how to do so for custom descriptors they might want to integrate into the dataset, we have provided in this feature insertion script an example use case that adds each of Expasy’s residue-level protein scale annotations for each residue type in the dataset. Moreover, included with the dataset’s source code is the ability for users to add any residue-level features currently available within the Graphein Python library (e.g., https://graphein.ai/modules/graphein.protein.html). As Graphein provides several different collections of useful residue-level features for users’ convenience, one can then add arbitrary Graphein features to each DIPS-Plus complex to significantly enhance the usability and extensibility of the dataset.

## Data Availability

Preprocessed data for DIPS-Plus^10^ as well as its associated source code and instructions for data processing and reproducibility can be found on Zenodo and GitHub, respectively. The GitHub instructions illustrate how users can install the Python programming language^46^ and build an Anaconda virtual environment^47^ containing the software dependencies required to preprocess and analyze DIPS-Plus^10^ using the provided Python scripts. Lastly, the GitHub instructions show users how to run such scripts and the order in which to do so to successfully rebuild DIPS-Plus^10^ from scratch, to featurize a given PDB file, or to train new machine learning models (e.g., NeiA) for protein interface prediction. For provenance, the original DIPS dataset’s source code^36^ can also be found on GitHub, along with a corresponding DOI.
